# Mental health among unaccompanied refugee minors after settling in Norway: A matched cross-sectional study

**DOI:** 10.1177/14034948221100103

**Published:** 2022-06-09

**Authors:** Sondre Aasen Nilsen, Ingrid Kvestad, Sølve Bjørn Randal, Mari Hysing, Nawar Sayyad, Tormod Bøe

**Affiliations:** 1 Regional Centre for Child and Youth Mental Health and Child Welfare, NORCE Norwegian Research Centre, Norway; 2 Child Welfare Services for Unaccompanied Refugee Minors, Bergen Municipality, Norway; 3 Department of Psychosocial Science, Faculty of Psychology, University of Bergen, Norway

**Keywords:** Unaccompanied refugee minors, mental health, SDQ, child welfare, adolescence

## Abstract

*Aims:* To describe the mental health of unaccompanied refugee minors (URMs) settled in Norway and compare their responses to an age- and sex-matched sample of Norwegian young people. *Methods:* The data were from the Pathways to Independence study of URMs aged 15–20 years (*n* = 81; 82.7% male; response rate 80%) conducted in 2018–2019 in the Bergen municipality, Norway. The data from the URMs were linked to an age- and sex-matched group of young people from the Norwegian youth@hordaland study conducted in 2012 (*n* = 324). Mental health was assessed by the Strengths and Difficulties Questionnaire (SDQ). *Results:* URMs were more likely to agree with most items pertaining to emotional problems, peer problems and prosocial subscales than Norwegian young people. Few differences were found for items on the conduct problems and hyperactivity-inattention problems scales. Poor psychometric properties, including weak factor loadings and low internal consistency, were detected for the SDQ subscales among URMs, except for the emotional problems subscale, indicating that the originally proposed five-factor model fitted the data poorly. ***Conclusions:* URMs appear to have moderately more emotional problems than Norwegian young people. They are more likely to report being alone, getting along better with adults than with their peers and being bullied, but also report being more helpful and sharing with others. Studies with larger samples of URMs should determine the most appropriate factor structure of the SDQ when administered to URM samples.**

## Introduction

About 90,000 unaccompanied refugee minors (URMs) arrived in Europe in 2015 [1]. The term URM refers to children younger than 18 years on arrival, without parents or legal guardians, who have fled their country of origin to seek protection and asylum in a new country [2]. During the recent migrant crises, many URMs have entered Europe by crossing the Mediterranean Sea to escape war, violence and persecution [3,4]. URMs have often been subjected to multiple traumatic events, including witnessing murder, violence, sexual assaults, loss of family and poverty [5,6]. These minors represent a heterogenous group who differ in their ethnicity, socioeconomic background, reasons for fleeing their home and subsequent needs upon arrival in their host country [7–9].

From 1996 to 2019, 18,229 young people applied for asylum as URMs in Norway, with the peak of applications received in 2015. In this period, 9943 young people were granted a residence permit and by the start of 2019, 9344 were still living in Norway. About 40% of these URMs were settled in Norwegian municipalities from 2014 to 2018. Most of the URMs were boys and older 15 years on arrival, arriving from Afghanistan, Eritrea, Syria and Somalia [10,11]. In Norway, it is primarily the Child Welfare Services (CWS) that have responsibility for placement and follow-ups [12]. Younger URMs are mostly placed in institutional care or in group homes with staff working 24-h shifts, whereas older URMs tend to live in homes that are partly staffed or in studio apartments where they receive follow-up as needed. Some also reside in foster care or in host families [13]. In Norway, URMs can be followed-up by the CWS until the age of 25 years.

Exposure to trauma, either in their country of origin or during flight, is prevalent among refugees, particularly among URMs [6,14,15]. These events may be especially harmful to URMs, who experience them during their formative years without the support of parents or guardians. A systematic review of studies of URMs in Europe found that they display high rates of mental health problems, exceeding the levels of minor refugees accompanied by their parents [16]. In particular, symptoms of post-traumatic stress disorder, anxiety and depression are common. For instance, a study from Belgium found that URMs scored about 1.0 and 1.7 standard deviation (SD) units higher on a measure of post-traumatic stress disorder and in the range of 0.6–1.0 SD units higher on measures of internalizing problems, such as anxiety and depression, than accompanied minor refugees and native young people, respectively [6]. Similarly, a study from Germany found that about 65% of URMs scored above the clinical cut-off for post-traumatic symptoms and 43 and 38% scored above the clinical cut-off for depression and anxiety [14].

High levels of internalizing problems in URMs have also been reported in previous studies from Norway [17,18] and one study found that internalizing problems remained high at five years of follow-up [19]. However, as noted in a recent review [20], there is great variability in the prevalence of mental health problems such as depression (13–76%) and anxiety (11–85%) among URMs. This variability probably stems from differences in the characteristics of the sample studied (e.g. age, country of origin and host country), assessment methods (e.g. self-report versus clinical assessment) and the follow-up received on arriving in a new country.

Externalizing mental health problems appear less consistently elevated in URMs [6,14,21]. Using the Strengths and Difficulties Questionnaire (SDQ) in a study from Italy, URMs reported significantly lower levels of conduct problems and more prosocial behaviours, but had more peer problems than native Italian young people. No significant difference was detected on the hyperactivity-inattention subscale [21]. Similarly, using the Hopkins Symptom Checklist, a study from the Netherlands found that URMs scored considerably lower than native young people on the externalizing problems subscale (corresponding to a Cohen’s *d* = 0.72) [6]. These findings suggest that an assessment of multiple psychological domains is needed to capture mental health among URMs.

Although high levels of mental health problems, particularly internalizing problems, have been reported in previous studies, little is known about URMs in Norway following the increased migration affecting Europe in 2015. Only one previous study has compared URMs with Norwegian young people and only regarding depression symptoms [17]. As rates of mental health problems among URMs differ widely across studies [20], there is a need for studies comparing a broader range of mental health domains among URMs in Norway to better identify their potential health service needs. Based on these considerations, this study sought to investigate mental health problems among URMs after settling in Norway compared with a reference group of sex- and age-matched young people in the general population of Norway.

## Methods

### Samples and procedures

The data for the URMs were from the Pathways to Independence study [22] conducted among URMs granted a residence permit and under the care of the CWS for unaccompanied refugee minors (URM CWS) in the Bergen municipality, Norway. The URM CWS coordinated the data collection, which lasted from December 2018 to January 2019 [22]. All the URMs in contact with the CWS in Bergen and aged 15 years or older were invited to participate. From the target population of 116 URMs, ten URMs were considered ineligible to participate as a result of evasive behaviour and poor mental health, three were excluded due to the inability of the CWS to provide the participants with proper follow-up after the survey (i.e. the participants were living in other parts of the country) and two were excluded due to the unavailability of the case worker. Hence the number of invited URMs was 101 and 81 consented and were included, yielding a participation rate of 80%. The URMs were provided with written and oral information about the study in advance and consented to participate by filling out a consent form on the first page of the online questionnaire after going through the information thoroughly with the case workers [22].

The online survey was in Norwegian because most of the questionnaires used in the study did not have official translated and validated versions for all the languages spoken by the URMs. A pilot study testing the questionnaire in older URMs previously under the care of the URM CWS suggested that the survey was feasible for most URMs when used in the Norwegian language [22]. The URMs completed the online survey at the case worker’s office. Case workers were present and available for questions and queries while the URMs filled in the questionnaire, but were instructed not to look at the participants’ responses. The case workers were mainly Norwegian adults with a background in social work employed at the CWS.

The SDQ was administered as a part of a longer survey including other instruments. Overall, the entire survey took about 1–2 h to complete for most participants. Interpreter services were made available for the few URMs (*n* = 6) who were unable to complete the survey in Norwegian. Case workers clarified words and sentences that the URMs found difficult to understand when no interpreter was present. After the URMs had completed the questionnaire, the case workers were instructed to be available for follow-up when needed. Procedures were made for referrals to further counselling outside the URM CWS in case of adverse reactions to the questionnaire.

### Reference group

We drew on data from the youth@hordaland study (*N* = 10,257, response rate = 53%) to obtain a relevant reference group of similarly aged Norwegian young people in the general population living in the same county as the URMs. The youth@hordaland study was a web-based, population-based survey of adolescents aged 16–19 years conducted in spring 2012 in Hordaland County, Norway. Bergen was the largest city in Hordaland County during the study and most participants in the youth@hordaland resided there. The main aim of the youth@hordaland study was to assess mental health and health service use during adolescence. Young people in school at the time of the study received study information and a link to participate by SMS and their school email address. The schools were encouraged to allocate one school hour for the young people to complete the questionnaire. Adolescents not enrolled in school were sent information by post to their home address. The adolescents could respond at their convenience (e.g. at home) throughout the data collection period. The entire survey took about 45 minutes to complete. The adolescents consented to participation electronically. The youth@hordaland has been thoroughly detailed elsewhere [23] and has also been used as a matched control group in previous publications [24,25].

For every participating URMs from the Pathways to Independence study, four sex- and age-matched young people from the youth@hordaland study (*n* = 324) were randomly selected to the reference group. To achieve a 1:4 ratio between cases and controls, we had to expand the eligible age range of matches to ±1.1 years. We matched on sex due to the skewed distribution of sex in the URM sample (17.3% girls) and because girls tend to report more internalizing mental health problems than males [26]. We also matched on age as mental health problems tend to increase during adolescence [27]. Although the youth@hordaland study and the Pathways to Independence study were conducted six years apart, participants from youth@hordaland were considered as the best available candidates for the reference group because the youth@hordaland study consists of a well-defined cohort of similarly aged adolescents residing in the same county as the URMs.

### Ethics

The Pathways to Independence study (approval number: 2018/966) and the youth@hordaland study (approval number: 2012/1467) were approved by the Regional Committee for Medical and Health Research Ethics of Western Norway and conducted following recommendations from the Norwegian Data Protection Services. Participation in the studies was voluntary and the participants could withdraw from the studies at any time. In both studies, participants aged 16 years or older consented to participate in accordance with Norwegian regulations. For URMs aged 15 years, consent was also obtained from their legal guardians.

### Measures

#### Sociodemographic information

In the Pathways to Independence study, information about age, sex and country of origin was obtained by adolescent self-report. Years since arrival was calculated by subtracting age at participation by age on arrival in Norway.

In the youth@hordaland study sex and date of birth were identified through the participants’ personal identity number in the Norwegian National Population Register. Exact age was estimated by calculating the interval of time between the date of birth and date of participation. Socioeconomic status was assessed both by perceived economic well-being and parental education. Perceived economic well-being was reported with three response options: ‘poorer than others’, ‘equal to others’ and ‘better than others.’ ’Parental education’ was rated by adolescent self-report using the options ‘elementary school’, ‘high school, vocational’, ‘high school, general’, ‘college/university, less than four years’, ‘college/university, four years or more’ or ‘don’t know’. The response options were collapsed into basic (elementary school level), intermediate (high school levels), higher (college/university levels) and unknown.

#### Mental health

In both studies, mental health problems were assessed by the SDQ [28]. The SDQ is a screening instrument for mental health problems among children and adolescents and consists of five subscales measuring emotional problems, conduct problems, hyperactivity-inattention problems, peer problems and prosocial behaviours. Each of the subscales consists of five items and the respondents indicated on a three-point Likert scale to which extent a symptom applied to them (i.e. ‘not true’, ‘somewhat true’ and ‘certainly true’). Summarizing the items gives a subscale score ranging from 0 to 10, with higher scores indicating more problems. The SDQ total score, as an overall measure of mental health problems, is created by summing the scores on all subscales, excluding the prosocial behaviour scale.

The psychometric properties of the SDQ are generally considered to be strong [29] and to have adequate reliability and validity among adolescents in Western countries [23,30]. However, various factor solutions of the SDQ has been supported [31–33] and the best-fitting model may be sample-dependent [34]. Previous investigations have found the original five-factor solution to also work adequately for older adolescents in the youth@hordaland study [23]. The utility of the SDQ when used by children and adolescents of refugee backgrounds have been questioned. A review found little support for the originally proposed five-factor model of the SDQ when used in languages spoken by children and adolescents of refugee backgrounds, suggesting that, for this group, the SDQ subscales may measure different constructs than among Western young people [35]. A similar conclusion was also reached by a study on minor refugees in Australia, finding poor psychometric properties, particularly for the prosocial (ceiling effects) and peer problems (low internal consistency) subscales [36].

### Statistical analyses

All analyses were performed using R version 4.0.2 for Windows [37]. Data preparations were conducted with packages and functions from the tidyverse [38]. As an initial step to inform our analytical strategy, we investigated the psychometric properties of the SDQ by performing a series of confirmatory factor analyses (CFAs). The purpose of these analyses was to examine whether the SDQ subscales were meaningful categories in the URM sample and thus whether reporting and comparing mean scores of the SDQ subscales in the URM sample and the sample from youth@hordaland were warranted.

It has been recommended that a sample with at least ten cases per parameter to be estimated is needed to perform a CFA [39]. It is also worth noting that sample size requirements depend on several factors, including the complexity of the model (e.g. number of latent variables, number of indicators and strength of factor loadings) and the presence of missing data [40]. Given this general rule of thumb and the complexity of the SDQ (i.e. five latent variables with five indicators each), our sample of URMs (*n* = 81) was too small to perform a CFA of the proposed five-factor solution for the SDQ and thus also too small to conduct any rigorous measurement invariance testing between the two samples on the five-factor solution of the SDQ.

To obtain a sense of how the SDQ performed on the two samples, we instead proceeded by defining each subscale as a unidimensional measurement scale (one latent factor with five indicators) and conducted a CFA on each of the subscales separately for the two samples. Model fit, factor loadings and internal consistency were then compared between the two groups. The robust-weighted least-squares estimator was used in the CFAs as a result of the skewed categorical data (ordinal data with three response options). The comparative fit index (CFI) and the root-mean-square error of approximation (RMSEA) were used to assess the model fit, with a CFI >0.95 and a RMSEA <0.06 signifying a good fit to the data [41].

We also calculated subscale correlations and item correlations within each subscale and report McDonald’s *ω* and Cronbach’s *α* as measures of internal consistency. McDonald’s *ω* does not assume TAU-equivalence as the *α* (i.e. equal factor loadings) and has been shown to be a more sensible index than Cronbach’s *α* in several circumstances [42]. The peer problems subscale resulted in negative variances in the URM sample and factor loadings could not be computed. As McDonald’s *ω* is partly based on factor loadings, it could not be calculated for this subscale. We therefore also report Cronbach’s *α* for all subscales.

As a result of the partly weak internal consistency measures and factor loadings across the SDQ subscales among the URMs, our primary analytical strategy was to describe the item responses among URMs and compare them with the item responses from the youth@hordaland study. We did this by calculating the standardized mean difference on each SDQ item between participants in the sample of URMs and youth@hordaland. The results are presented as a forest plot created with the R package meta [43]. To facilitate comparison with the existing literature, we also present the overall subscale effects created by calculating the standardized differences (Hedges’ g) between the pooled means of items within each SDQ subscale using the R package effsize [44]. As the items within the peer problems subscale among URMs hardly correlated (*α* = 0.18) and no measurement model could be properly identified using CFA, we do not report any overall subscale score for this measure. By the same rationale, we removed the peer problems subscale from the calculation of the overall SDQ score (SDQ total score). A ridge plot [45] was created to visualize the distribution of symptom scores in the two samples.

Only one respondent in the URM group had missing values on the SDQ items and was removed from the analyses.

## Results

### Sociodemographic characteristics

Table I give the demographic information for the two samples. The mean (M) age of the URMS (M = 18.00, standard deviation [SD] = 1.33) was slightly higher than that of the reference group (M = 17.81, SD = 0.96) and 17.3% were females. Most of the URMs came from Afghanistan (47%), followed by Eritrea, Syria and Somalia. The mean time since arrival in Norway was 3.5 years (SD = 2.2). Most young people from the youth@hordaland study were Norwegian, had parents with intermediate or higher educational qualifications and perceived their family’s economic well-being as ‘equal to most others’.

**Table I.** Sociodemographic characteristics of the Pathways to Independence study (*N* = 81) and the matched control group from the youth@hordaland study (*N* = 324).

	URMs (*N* = 81)	Matched control group (y@h) (*N* = 324)
**Age (years)**	18.00 (1.33)|(15–20)	17.81 (0.96)|(16–19)
**Female sex**	14 (17.3)	56 (17.3)
**Time since arrival (years)**	3.5 (2.2)	
**Homeland**		
Afghanistan	38 (46.9)	–
Albania	8 (9.9)	–
Eritrea	14 (17.3)	–
Somalia	7 (8.6)	–
Syria	14 (17.3)	–
**Ethnicity (y@h)**		
Foreign-born	–	19 (6.0)
**Maternal education (y@h)**		
Basic	–	29 (9.0)
Intermediate	–	83 (25.8)
High	–	146 (45.3)
Unknown	–	64 (19.9)
**Paternal education (y@h)**		
Basic	–	32 (10)
Intermediate	–	108 (33.6)
High	–	105 (32.7)
Unknown	–	76 (23.7)
**PEW (y@h)**		
Worse than others	–	32 (10.2)
Equal to most others	–	213 (67.6)
Better than others	–	70 (22.2)

Data presented as *n* (%) or Mean (SD) (range) values.

URMs: unaccompanied refugee minors; matched control group (y@h): matched control group from the youth@hordaland study; ethnicity y@h reference: Norwegian; PEW: perceived economic well-being.

#### Item responses and psychometric investigations of the SDQ

Table II gives the item responses and subscale scores on the SDQ for the URMs and the reference group. The URMs were more likely to agree with items on the peer problems and prosocial subscale than the reference group

**Table II.** Item responses on the Strengths and Difficulties Questionnaire (SDQ) and mean SDQ subscale and total scores among unaccompanied refugee minors from the Pathways of Independence study (*N* = 81) and the matched control group from the youth@hordaland study (*N* = 324).

	URMs (*N* = 81)			Matched control group (y@h) (*N* = 324)		
	Not true	Somewhat true	Certainly true	Not true	Somewhat true	Certainly true
**Emotion**						
Somatic	53.09	30.86	16.05	45.99	35.19	18.83
Worries	13.75	46.25	40.00	30.56	45.99	23.46
Unhappy	40.74	45.68	13.58	55.86	35.19	8.95
Clingy	17.28	43.21	39.51	30.86	45.06	24.07
Afraid	48.75	38.75	12.50	67.90	22.84	9.26
*Subscale*						
Mean (SD)	4.47 (2.20)			3.53 (2.52)		
Range	0–10			0–10		
**Conduct**						
Tantrum	50.62	38.27	11.11	52.16	37.65	10.19
Obeys^a^	1.23	33.33	65.43	2.16	45.06	52.78
Fights	96.30	2.47	1.23	94.44	4.32	1.23
Lies	65.00	22.50	12.50	89.20	8.02	2.78
Steals	86.25	10.00	3.75	93.52	4.32	2.16
*Subscale*						
Mean (SD)	1.67 (1.44)			1.36 (1.37)		
Range	0–6			0–8		
**Hyper**						
Restless	36.25	37.50	26.25	31.48	54.94	13.58
Fidgety	37.97	40.51	21.52	46.91	44.75	8.33
Distract	27.50	51.25	21.25	29.94	45.68	24.38
Reflect^a^	10.00	38.75	51.25	4.94	60.49	34.57
Attends^a^	6.25	51.25	42.50	12.96	59.88	27.16
*Subscale*						
Mean (SD)	3.91 (2.05)			3.94 (2.16)		
Range	0–9			0–9		
**Peer**						
Loner	12.50	43.75	43.75	50.62	39.51	9.88
Friend^a^	6.25	21.25	72.50	1.54	8.02	90.43
Popular^a^	2.50	47.50	50.00	2.78	39.81	57.41
Bullied	76.54	12.35	11.11	93.52	4.63	1.85
Oldbest	16.25	48.75	35.00	62.65	28.70	8.64
*Subscale*						
Mean (SD)	3.71 (1.55)			1.7 (1.67)		
Range	1–9			0–9		
**Prosocial**						
Considerate	0.00	7.41	92.59	0.93	12.65	86.42
Shares	1.23	25.93	72.84	3.40	37.96	58.64
Caring	3.70	23.46	72.84	2.47	22.53	75.00
Kind	3.75	10.00	86.25	3.70	23.77	72.53
Helpout	3.75	36.25	60.00	8.33	59.57	32.10
*Subscale*						
Mean (SD)	8.72 (1.37)			8.06 (1.62)		
Range	4–10			1–10		
**SDQ total score**						
Mean (SD)	13.80 (4.83)			10.54 (5.35)		
Range	2–27			0–30		

Data presented as percentages or Mean (SD) and range.

a Positively worded items.

URMs: unaccompanied refugee minors; matched control group (y@h): matched control group from the youth@hordaland study.

The results from the psychometric investigations of the SDQ are shown in Supplemental Table I and Supplemental Figure 1, available online. In brief, fit indices suggested that the emotional problems, conduct problems and prosocial subscales had good fits to the data across both samples. However, the item correlation matrix showed that many items within each subscale were weakly correlated (*r* < 0.2) among the URMs and the internal consistency reliability was poor (both *α* and *ω* < 0.6) across all subscales, except for emotional problems (*α* = 0.60, *ω* = 0.62). This was particularly the case for the peer problems subscale, where the strongest correlation (*r* = 0.22) was found between the items ‘popular’ (‘generally liked by others’) and ‘friend’ (‘has one good friend or more’), whereas the remaining item correlations were *r* < 0.13, resulting in a Cronbach’s *α* of 0.18.

**Figure 1.**
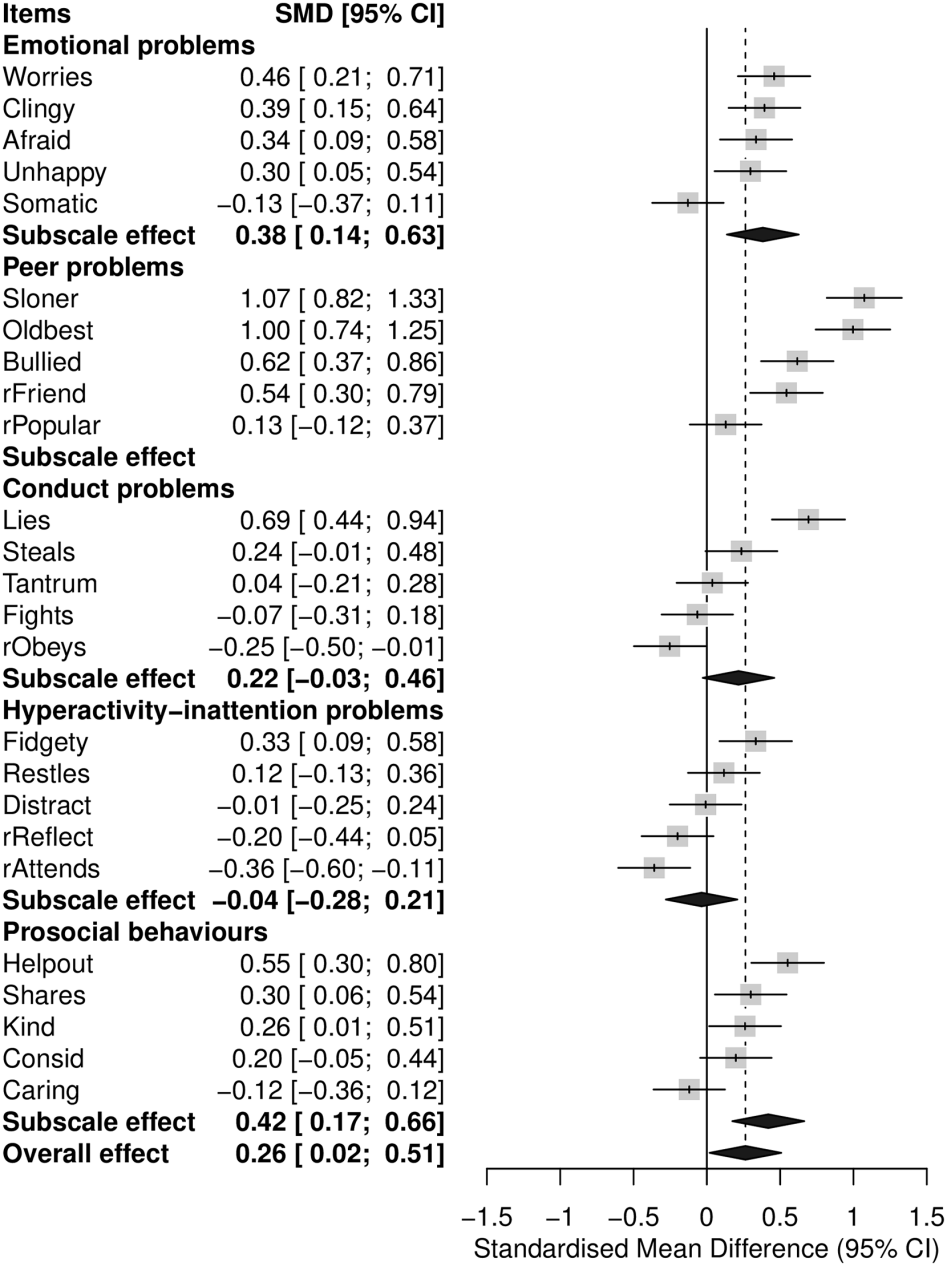
Forest plot of SDQ item and subscale scores comparing the URMs (*N* = 81) with a matched control group of young people from the youth@hordaland study (*N* = 324). SMD (95% CI) = standardized mean differences (Hedges’ g) with 95% confidence intervals. Scores to the right of the solid vertical line indicate that the URMs’ scores are higher than the adolescents from the youth@hordaland study. Items denoted “r” are positively worded items that have been reversed so that a higher score represents more problems.

Several item factor loadings were observably different between the two samples across the subscales and generally weaker among the URMs than in the reference group. Most notably, the ‘obeys’ item (‘usually does as told’) hardly loaded on the latent conduct problems factor among the URMs (standardized factor loading [λ] = 0.032). Weak factor loadings were also observed for the ‘reflective’ (‘thinks before doing things’; λ = 0.168) item on the hyperactivity subscale and the item ‘kind’ (‘kind to younger children’; λ = 0.249) on the prosocial subscale. The hyperactivity-inattention subscale had poor fit across both samples. Correlations between the SDQ subscales also differed between the samples (see Supplemental materials, available online, for details). Overall, these results indicate that measurement invariance cannot be assumed for the SDQ subscales across the two samples.

#### Comparison between the URMs and Norwegian young people on the SDQ on the SDQ

Figure 1 shows the SDQ item, subscale and total score (denoted overall effect) differences between the URMs and the reference group. On the subscale item level, the URMs had higher mean scores on all items except for the ‘somatic’ item (‘get a lot of headaches, stomach-aches or sickness’) on the emotional problems subscale. On the peer problems subscale, the URMs had particularity higher mean scores on items the ‘loner’ (‘rather alone than with people of their age’) and ‘oldbest’ (‘gets better along with adults than peers’). They were also more likely to agree with the ‘bullied’ item (‘often bullied’) and less likely to agree with the ‘friend’ item (‘has one good friend or more’) than the reference group.

No mean difference was detected for any items on the conduct problems subscale, except for the ‘lies’ item (‘often accused of lying or cheating’). On the hyperactivity-inattention subscale, URMs had a higher mean score on the ‘fidgety’ item (‘constantly fidgeting or squirming’) and a lower mean score on the ‘attends’ item (‘has good attention’). The URMs had significantly higher mean scores on the prosocial subscale items ‘helpout*’* (‘often offer to help others’), shares (‘shares readily with others’) and ‘kind’ (‘kind to younger children’) than the reference group.

**Figure 2.**
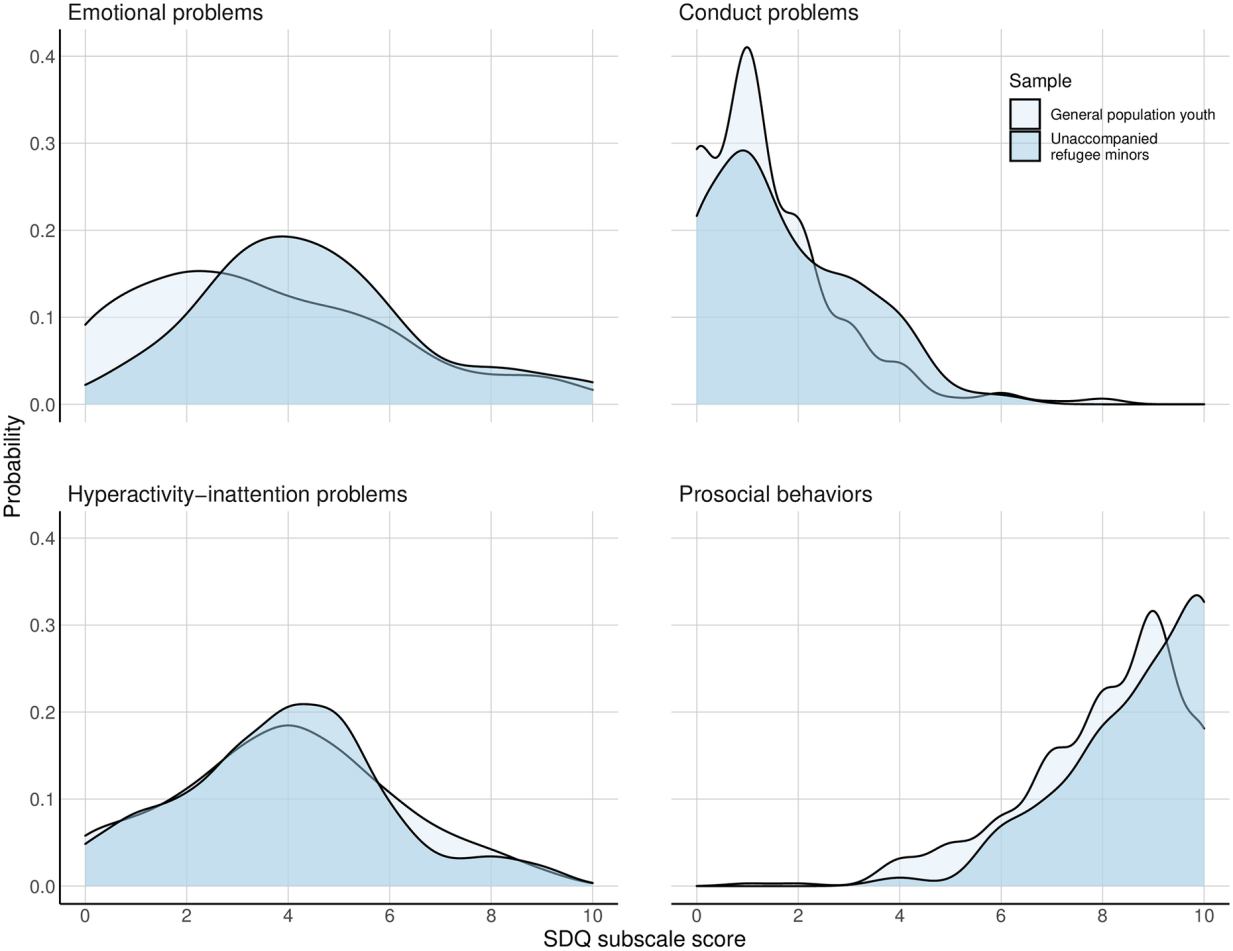
Ridge plot showing the probability density distribution of the SDQ subscale scores among the URMs (*N* = 81) (dark blue) and the matched control group of young people in the general population from the youth@hordaland study (*N* = 324) (light blue).

On the subscale score level, the URMs scored significantly higher on the emotional problems (Hedge’s *g* = 0.38) and prosocial subscales (Hedge’s *g* = 0.42) than the reference group. Indications of a ceiling effect was observed for the prosocial subscale, particularly among the URMs (Figure 2). The overall effect corresponded to a Hedge’s *g* of 0.26 (95% CI 0.02–0.51). If including the peer problems subscale, the overall effect was larger (Hedge’s *g* = 0.59 (95% CI 0.35–0.84)).

## Discussion

This study sought to examine mental health problems among a cohort of URMs cared for by the Norwegian CWS and to compare their responses to a reference group of age- and sex-matched young people from a population-based sample. Overall, the URMs scored significantly higher on most items pertaining to the emotional and peer problems subscales, and for some items on the prosocial subscale, than their peers from the general population. Few differences were detected for items on the conduct problems and hyperactivity-inattention problems scales. However, except for the emotional problems subscale, weak psychometric properties were detected in all SDQ subscales among the URMs, indicating that the original five-factor model does not provide the best fit to these data among the URMs, suggesting that the findings should be interpreted cautiously.

The URMs scored higher than reference group on most items intended to assess internalizing symptoms, although fewer differences were detected for items intended to measure externalizing symptoms. Overall, these findings align with previous findings suggesting internalizing problems to be more prevalent than externalizing problems in this group [6,21,46]. For instance, a study from Italy reported a similar pattern whereby URMs were more likely to score above the ‘borderline’ and ‘abnormal’ cut-off scores on the SDQ emotional problems scale than their native Italian peers, but were not more likely to score above set cut-offs on the conduct and hyperactivity-inattention scales. The emotional problems scale emerged as the most psychometrically sound SDQ subscale among the URMs in our study. Combined with the well-established links between traumatic experiences and symptoms of anxiety and depression [47], we find it likely that the present finding reflects that URMs are particularly vulnerable to such symptoms.

The URMs were more likely to agree with items pertaining to the peer problems subscale than the reference group. They were more likely to report that they preferred to be alone than with people of their age and that they got along better with adults than their peers. They were also, but to a lesser extent, more prone to report being bullied and to not have one or more good friends. Similar findings have been reported previously and have been interpreted as reflecting ‘acculturation difficulties’ or due to the URMs adopting a ‘adultomorphic’ behaviour by, for instance, working to help their family of origin economically [21]. High rates of experienced bullying among the URMs are also in accordance with a Swedish study [48]. Elevated scores among the URMs on these items might indeed reflect real struggles to be accepted by, and form meaningful bonds with, their native peers on arrival in a new country.

In parallel, ‘peer problems’ did not emerge as a meaningful construct following the psychometric investigations in this study. Inter-item correlations were weak and no measurement model was properly identified following a CFA. Previous research on the SDQ in languages spoken by children and adolescents of refugee backgrounds have also found poor factor structure and internal consistency of the peer problems subscale [35]. Comparable findings were reported by a study on minor refugees in Australia [36], which also noted that the item ‘gets better along with adults than peers’ was particularly problematic, as in our study. One reason for the poor properties of this subscale could be due to cultural variations in response styles or conceptions of problematic behaviour [35]. For instance, the ‘getting along with older people’ item could, in some cultures, actually be perceived as something positive [36] and may perhaps also reflect a necessary coping strategy used by the URMs to adapt to their new host country.

The URMs scored significantly higher on three of five items on the prosocial subscale than the reference group. High scores on the prosocial subscale of the SDQ among immigrant and refugee populations have been reported previously [21,36], although some studies were unable to find differences when compared with native young people [49,50]. Exposure to adverse events such as violence, terror and war has undoubtedly been linked to a host of negative outcomes. However, it has also been linked to post-traumatic growth and altruistic behaviour, also coined ‘altruism born of suffering’ [51]. For instance, high scores on the item ‘often offer to help others’ could reflect that URMs are motivated to help others due to the adverse experiences in their own lives [21]. On the subscale level, a notable ceiling effect was observed whereby most respondents scored at the higher end of the scale (median value nine of a total of 10). Ceiling effects on the prosocial subscale have also been reported in other studies on refugee populations[35,36]. This might indicate that the answers of the URMs to these items are influenced by demand characteristics or social desirability, which should be kept in mind when interpreting their responses to these items.

The relevance of our findings of poor construct validity of the SDQ in this cohort of URMs underscores the need to conduct more detailed psychometric investigations into the utility of screening tools in general, and the SDQ in particular, as used in larger samples of URMs. Similarly, although we cannot question the validity of the URMs’ answers item by item, care should be made when using the originally proposed SDQ subscale scores as indicative of measuring an underlying latent construct (e.g. ‘peer problems’).

## Strengths and limitations

A strength of the current study was the relatively high participation rate among the URMs (80%), a population that is generally considered hard to reach. Another strength was the age- and sex-matched reference group of young people from the large population-based youth@hordaland study.

Several limitations should be acknowledged. First, a notable weakness was the relatively small sample of URMs and the few girls, which prevented further subgroup analyses (e.g. by sex or country of origin). The small sample of URMs also restricted our psychometric investigations of the SDQ and have likely introduced some uncertainty in the presented estimates, which should therefore be interpreted with caution.

Second, the SDQ was administered in the Norwegian language. Interpreter services were made available for the few URMs (*n* = 6) who were unable to complete the survey in Norwegian and case workers were present during completion of the survey to clarify questions or words that the URMs found unclear. However, we cannot exclude that these contextual factors have had an impact on the responses of the URMs to the SDQ. However, our results mirror larger scale studies of similar samples, which lends some credibility to the results regarding the psychometric properties of the SDQ.

Third, the only variables available for matching were the age and sex of the participants from both samples. We recommend that the robustness of our findings should be assessed by replication in future studies where a richer set of variables for matching are available.

Fourth, it should be stressed that, due to the design of the present study, the differences detected between the URMs and Norwegian young people cannot causally be attributed to the URM status per se. This study is therefore descriptive in nature. To learn more about the adjustment of URMs, longitudinal studies comparing them with their peers from their country of origin not seeking refuge could further expand our knowledge of their adjustment on arrival in a new country.

Fifth, whereas the URM sample was assessed in 2018–2019, the youth@hordaland was conducted in 2012. We cannot exclude the possibility that differences in the time frame between these studies may have had some influence on the results. For instance, there are indications that rates of mental health problems among Norwegian adolescents have increased in recent years [52]. However, these changes are generally considered to be small and to be most prominent among older adolescent girls. As the samples used in the present study consist of mostly boys (83%), we do not believe that this has had any major impact on our results.

## Conclusions and implications

Our study found that the URMs reported moderately higher levels of emotional problems than a sample age- and sex-matched young people from the same geographical area in Norway. This finding highlights the need for the service providers, school staff and health personnel involved with URMs to be aware of emotional symptoms, such as anxiety and depression, among URMs. The URMs also had high agreement with several items on the peer problems and prosocial subscales. Their answers to these items might indicate that, although URMs perceive themselves to be kind and helpful to others, they struggle to form bonds with their peers in the host country. Combined, these results suggest that capturing their individual mental health and social needs may be important among health professionals and social workers involved with URMs. As these young people are on the verge of entering adulthood, the dilemmas of this transition should also be addressed and health professionals and policy-makers should be aware of the likely continued challenges faced by many of these young people after becoming adult members of society.

Indications of the weak construct validity of the SDQ subscales stress the need to be cautious when interpreting the subscale scores of URMs. Previous investigations of the factor structure of the SDQ on refugee populations have found little support for the original five-factor model [35,36]. Studies with larger samples of URMs should determine the most appropriate factor structure of the SDQ when administered to samples of URMs. As the best-fit structure may be sample-dependent, it may be more appropriate to interpret differences between the URMs and Western young people on the item rather than the subscale level. We also encourage researchers to be aware of the potential cultural influence on how these items are perceived. As such, this study supports the conclusion reached by other recent studies [35,36] that the SDQ should be used with caution as a screening instrument for mental health problems among URMs and that naïve comparisons of subscale means between refugee and general population samples should be avoided when using the SDQ.

## Supplemental Material

sj-docx-1-sjp-10.1177_14034948221100103 – Supplemental material for Mental health among unaccompanied refugee minors after settling in Norway: A matched cross-sectional study Click here for additional data file. Supplemental material, sj-docx-1-sjp-10.1177_14034948221100103 for Mental health among unaccompanied refugee minors after settling in Norway: A matched cross-sectional study by Sondre Aasen Nilsen, Ingrid Kvestad, S�lve Bj�rn Randal, Mari Hysing, Nawar Sayyad and Tormod B�e in Scandinavian Journal of Public Health

sj-tif-2-sjp-10.1177_14034948221100103 – Supplemental material for Mental health among unaccompanied refugee minors after settling in Norway: A matched cross-sectional study Click here for additional data file. Supplemental material, sj-tif-2-sjp-10.1177_14034948221100103 for Mental health among unaccompanied refugee minors after settling in Norway: A matched cross-sectional study by Sondre Aasen Nilsen, Ingrid Kvestad, S�lve Bj�rn Randal, Mari Hysing, Nawar Sayyad and Tormod B�e in Scandinavian Journal of Public Health
