# Supplementary material for: Mental health among unaccompanied refugee minors after settling in Norway: A matched cross-sectional study
Source: Scand J Public Health. 2022 Jun 9;51(3):430–41. doi: 10.1177/14034948221100103 (PMC10251457; doi:10.1177/14034948221100103)
Supplement: sj-docx-1-sjp-10.1177_14034948221100103 – Supplemental material for Mental health among unaccompanied refugee minors after settling in Norway: A matched cross-sectional study [file sj-docx-1-sjp-10.1177_14034948221100103.docx]

**Supplementary Table 1.** Fit indices, factor loadings, and item correlations for the SDQ-subscales by sample

|  | URMs | |  | youth@hordaland | |  |  |  |  |  |  |
| --- | --- | --- | --- | --- | --- | --- | --- | --- | --- | --- | --- |
|  | Standardized factor loadings (λ) | |  | Standardized factor loadings (λ) | |  | Item correlations (URMs below diagonal) | | | | |
| **Emotional problems** |  |  |  |  |  |  | Somatic | Worries | Unhappy | Clingy | Afraid |
| Somatic | 0.390 |  |  | 0.691 |  |  | x | 0.416 | 0.440 | 0.331 | 0.351 |
| Worries | 0.654 |  |  | 0.757 |  |  | 0.162 | x | 0.498 | 0.317 | 0.390 |
| Unhappy | 0.876 |  |  | 0.790 |  |  | 0.279 | 0.467 | x | 0.292 | 0.410 |
| Clingy | 0.342 |  |  | 0.565 |  |  | 0.027 | 0.242 | 0.164 | x | 0.371 |
| Afraid | 0.515 |  |  | 0.724 |  |  | 0.229 | 0.174 | 0.352 | 0.255 | x |
| Fit indices | CFI = 0.970, TIL = 0.940, RMSEA = 0.066 | | | CFI = 0.992, TIL = 0.984, RMSEA = 0.056 | | |  |  |  |  |  |
| Internal consistency | ω= 0.62, α = 0.60 |  |  | ω = 0.76 α = 0.71 |  |  |  |  |  |  |  |
|  |  |  |  |  |  |  | Item correlations (URMs below diagonal) | | | | |
| **Peer problems** |  |  |  |  |  |  | Loner | Friend | Popular | Bullied | Oldbest |
| Loner | - |  |  | 0.651 |  |  | x | 0.332 | 0.312 | 0.180 | 0.271 |
| Friend | - |  |  | 0.881 |  |  | 0.067 | x | 0.322 | 0.331 | 0.230 |
| Popular | - |  |  | 0.680 |  |  | -0.132 | 0.220 | x | 0.195 | 0.314 |
| Bullied | - |  |  | 0.564 |  |  | 0.078 | 0.077 | 0.038 | x | 0.149 |
| Oldbest | - |  |  | 0.559 |  |  | -0.053 | 0.119 | -0.066 | 0.018 | x |
| Fit indices | - |  |  | CFI = 1.000, TIL = 1.012, RMSEA = 0.000 | | |  |  |  |  |  |
| Internal consistency | α = 0.18 |  |  | ω = 0.65, α = 0.64 |  |  |  |  |  |  |  |
|  |  |  |  |  |  |  | Item correlations (URMs below diagonal) | | | | |
| **Conduct problems** |  |  |  |  |  |  | Tantrum | Obeys | Fights | Lies | Steals |
| Tantrum | 0.306 |  |  | 0.429 |  |  | x | 0.121 | 0.190 | 0.149 | 0.195 |
| Obeys | 0.032 |  |  | 0.446 |  |  | 0.037 | x | 0.195 | 0.301 | 0.052 |
| Fights | 0.872 |  |  | 0.907 |  |  | 0.085 | 0.090 | x | 0.301 | 0.390 |
| Lies | 0.853 |  |  | 0.689 |  |  | 0.234 | -0.080 | 0.268 | x | 0.322 |
| Steals | 0.349 |  |  | 0.714 |  |  | 0.052 | 0.151 | 0.050 | 0.102 | x |
| Fit indices | CFI = 1.000. TIL = 1.141, RMSEA = 0.000 | | | CFI = 0.986, TIL = 0.971, RMSEA = 0.034 | | |  |  |  |  |  |
| Internal consistency | ω = 0.45, α = 0.34 |  |  | ω= 0.53, α = 0.57 |  |  |  |  |  |  |  |
|  |  |  |  |  |  |  |  |  |  |  |  |
| **Hyperactivity problems** |  |  |  |  |  |  | Restless | Fidgety | Distract | Reflective | Attends |
| Restless | 0.639 |  |  | 0.667 |  |  | x | 0.508 | 0.375 | 0.257 | 0.222 |
| Fidgety | 0.406 |  |  | 0.647 |  |  | 0.330 | x | 0.344 | 0.157 | 0.214 |
| Distract | 0.711 |  |  | 0.880 |  |  | 0.360 | 0.344 | x | 0.247 | 0.595 |
| Reflective | 0.168 |  |  | 0.388 |  |  | -0.023 | 0.157 | 0.247 | x | 0.193 |
| Attends | 0.531 |  |  | 0.728 |  |  | 0.173 | 0.214 | 0.595 | 0.193 | x |
| Fit indices | CFI = 0.814, TIL = 0.628, RMSEA = 0.130 | | | CFI = 0.890, TIL = 0.780, RMSEA = 0.223 | | |  |  |  |  |  |
| Modified model^a^: | CFI = 1.000, TLI = 1.157, RMSEA = 0.000. | | | CFI = 0.999, TLI = 0.995, RMSEA = 0.033 | | |  |  |  |  |  |
| Internal consistency | ω = 0.57, α = 0.51 |  |  | ω= 0.79, α = 0.69 |  |  |  |  |  |  |  |
|  |  |  |  |  |  |  |  |  |  |  |  |
| **Prosocial** |  |  |  |  |  |  | Considerate | Shares | Caring | Kind | Helpout |
| Considerate | 0.621 |  |  | 0.718 |  |  | x | 0.262 | 0.411 | 0.264 | 0.130 |
| Shares | 0.523 |  |  | 0.584 |  |  | 0.124 | x | 0.366 | 0.223 | 0.135 |
| Caring | 0.636 |  |  | 0.873 |  |  | 0.105 | 0.246 | x | 0.360 | 0.191 |
| Kind | 0.249 |  |  | 0.611 |  |  | 0.123 | 0.094 | 0.020 | x | 0.175 |
| Helpout | 0.729 |  |  | 0.351 |  |  | 0.281 | 0.223 | 0.306 | 0.087 | x |
| Fit indices | CFI = 1.000, TIL = 1.193, RMSEA = 0.000 | | | CFI = 1.000, TIL = 1.029, RMSEA = 0.000, | | |  |  |  |  |  |
| Internal consistency | ω = 0.54, α = 0.49 |  |  | ω = 0.63, α = 0.63 |  |  |  |  |  |  |  |
| *Note. URMs = Unaccompanied refugee minors, ω*= McDonald’s omega, *α = Cronbach's alpha, CFI = Comparative fit index, RMSEA = Root mean square error of approximation, TLI = Tucker-Lewis index.* No measurement model could be identified for the Peer problems subscale for URMs.  *^a^The modified model among URMs allowed correlated error terms between the items “restles” and “fidgety, and “reflect” and “attend”, while the modified model in the youth@hordaland sample allowed correlated error terms between the items “restles” and “fidgety”, and “distract” and “attends”.* | | | | | | | | | | | |
