# Supplementary figures and images for: Mental health among unaccompanied refugee minors after settling in Norway: A matched cross-sectional study
Source: Scand J Public Health. 2022 Jun 9;51(3):430–41. doi: 10.1177/14034948221100103 (PMC10251457; doi:10.1177/14034948221100103)

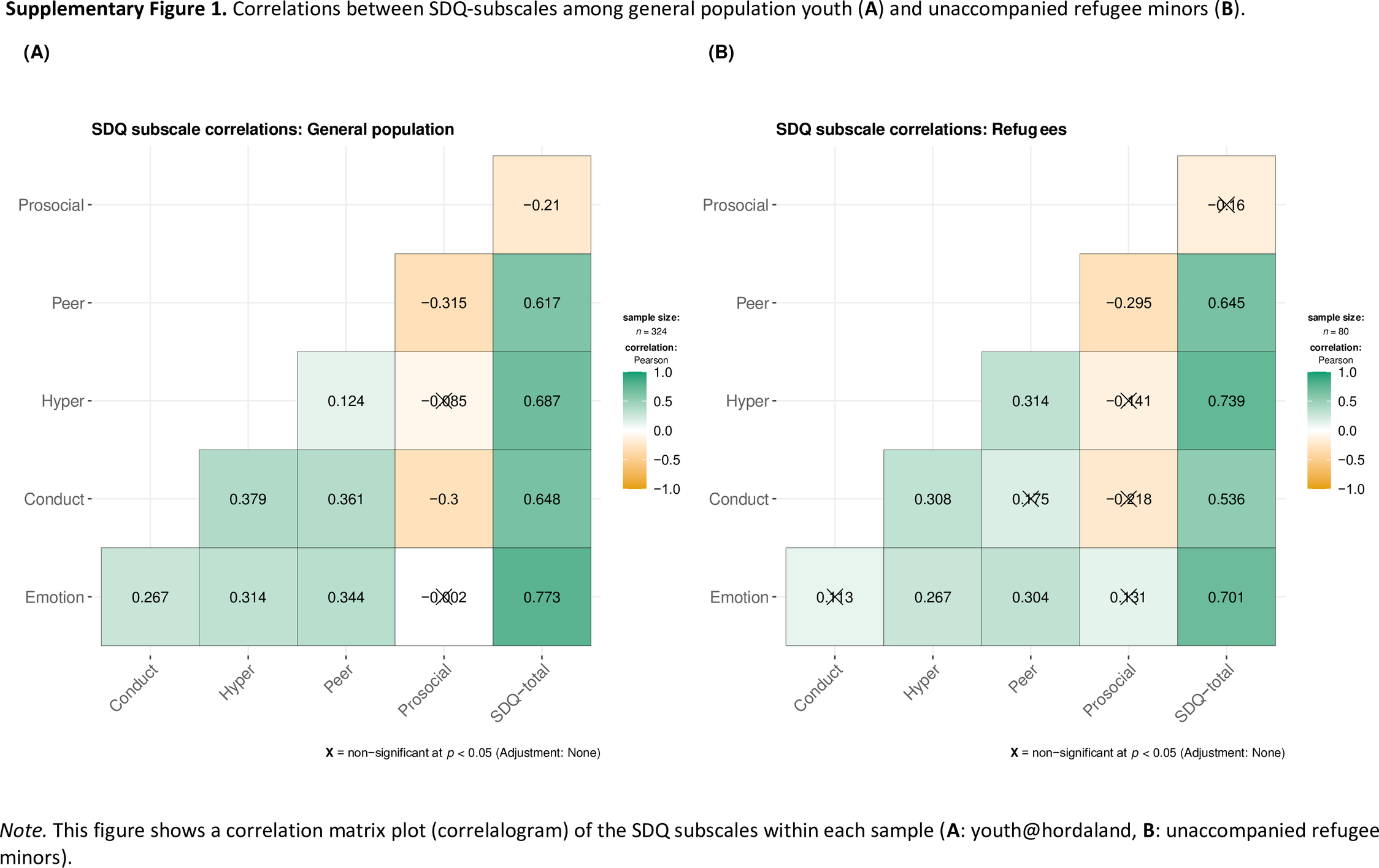

Supplement: sj-tif-2-sjp-10.1177_14034948221100103 – Supplemental material for Mental health among unaccompanied refugee minors after settling in Norway: A matched cross-sectional study [file sj-tif-2-sjp-10.1177_14034948221100103.tif]
